# Shock Wave Intravascular Lithotripsy (IVL)-Assisted Staged Percutaneous Coronary Intervention (PCI) for a Calcified Right Coronary Artery in a Patient With Unstable Angina: Shock the Rock

**DOI:** 10.7759/cureus.24489

**Published:** 2022-04-26

**Authors:** Sanjay Kumar Sharma

**Affiliations:** 1 Department of Cardiology, Neo Hospital, Noida, IND

**Keywords:** single-vessel disease, right coronary artery, percutaneous coronary intervention, lithotripsy, calcifications

## Abstract

Coronary artery calcification represents one of the challenging and demanding subsets of percutaneous coronary intervention (PCI). Accumulated evidence has aptly outlined that shockwave intravascular lithotripsy (IVL) is a reliable tool to overcome calcified stenosis in coronary arteries. However, there is a lack of published case reports in the Indian context. Herein, we describe a case of right coronary artery (RCA) calcifications successfully managed with shock wave IVL-assisted staged PCI. Initially, a 74-year-old male patient presented with ST-segment elevation myocardial infarction (STEMI). At that time, coronary angiography demonstrated calcific thrombotic occlusion in the left anterior descending artery (LAD) and stenosis in proximal and mid tubular RCA. It was decided to proceed with immediate PCI of LAD followed by staged PCI of RCA. The patient presented with unstable angina at the time of the second repeat PCI of RCA and was managed with shock wave IVL-assisted staged PCI. Ultimately, the patient’s condition was improved with good thrombolysis in myocardial infarction (TIMI) flow.

## Introduction

Coronary artery calcification (CAC) hinders percutaneous coronary intervention (PCI) by impeding stent crossing, disrupting drug-polymer from the stent surface, altering drug delivery and elution kinetics, and reducing stent expansion and apposition [1]. Thankful to the novel shockwave intravascular lithotripsy (IVL) system that emits circumferential mechanical energy and thus disrupts the calcium, minimizes soft-tissue injury while aiding stent deployment has greatly enriched therapeutic armamentarium of calcified lesions [2]. Despite the overwhelming clinical evidence with encouraging outcomes available for shock wave IVL-assisted PCI [3-5], there is a paucity of the data in the Indian context. Toward this end, we hereby report a case of a male patient with right coronary artery (RCA) calcifications presented with unstable angina. He was treated with shockwave IVL-assisted staged PCI.

## Case presentation

**Figure 1:**
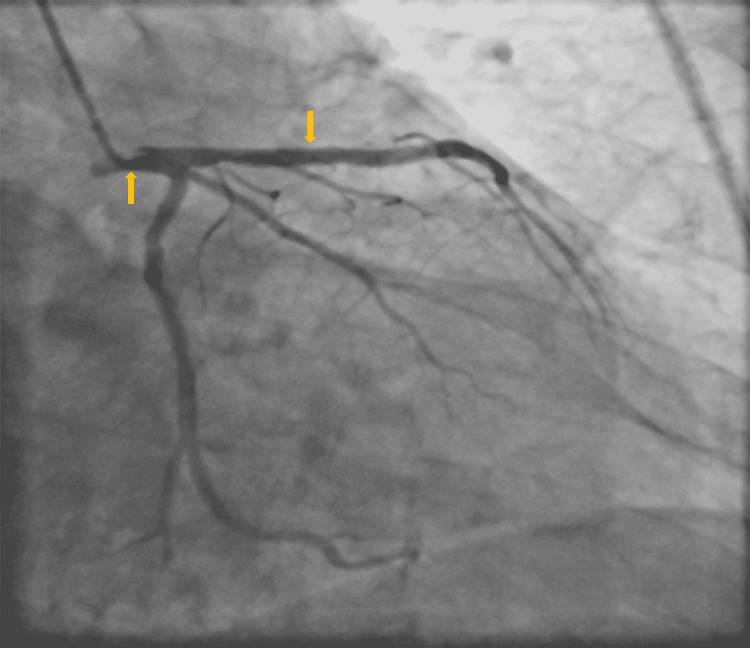
Normal left main, previously patent LAD stent LAD: left anterior descending artery

A 74-year-old male with a past history of hypertension, uncontrolled diabetes mellitus, and pancreatitis admitted to a tertiary care facility for staged PCI of RCA. He had developed ST-segment elevation myocardial infarction (STEMI) nearly one month ago, which was managed with the implantation of a drug-eluting stent (DES) (Figure 1).

**Figure 2:**
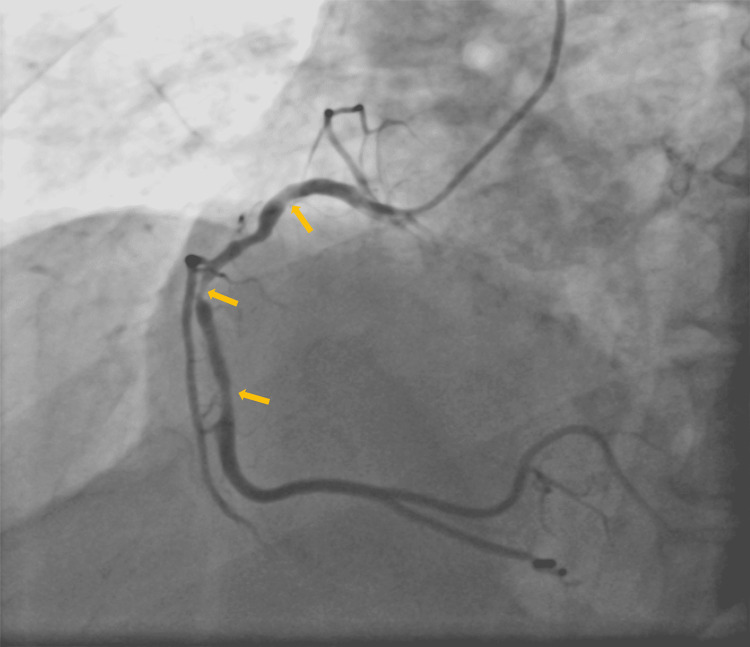
Coronary angiography of RCA revealed 60–70% stenosis—mild 95% calcific stenosis followed by 70% diffuse stenosis, normal PDA/PLV PDA: posterior descending artery; PLV: posterior left ventricular; RCA: ight coronary artery

At that time, transthoracic echocardiography revealed akinetic mid anterior septum, apex, mid anterior wall, dilated left ventricle, severe left ventricular systolic dysfunction with an ejection fraction of 30%, grade 1 diastolic dysfunction, and mild aortic regurgitation. All laboratory parameters were normal, except for troponin-I (619.9 ng/dl). Further, coronary angiography revealed normal left main (LM), 100% calcific thrombotic stenosis in left anterior descending artery (LAD), 50% stenosis in proximal left circumflex artery (LCX), and normal obtuse marginal artery-OM1 and OM2, 70% stenosis in proximal RCA and 80-90% stenosis in mid tubular RCA, normal posterior descending artery (PDA)/posterior left ventricular (PLV) artery. We planned to proceed with the immediate PCI of LAD followed by staged PCI of RCA. Nearly one month later, the patient was admitted to an intensive care unit (ICU) for the second PCI using shockwave IVL system. At this time, he was presented with unstable angina. On admission, the patient had a blood pressure of 143/86 mmHg, a pulse rate of 69 beats per minute, and oxygen saturation of 99% on room air. Moreover, chest and neurological examinations were normal. Coronary angiography was performed at arrival demonstrated normal LM, type-II vessel proximal to mild patent stent in LAD, normal diagonal arteries-D1 and D2, 40-50% stenosis in proximal LCX, normal OM1 and OM2 arteries, 60-70% stenosis in RCA-mild 95% calcific stenosis followed by 70% diffuse stenosis, normal PDA/PLV (Figure 2).

**Figure 3:**
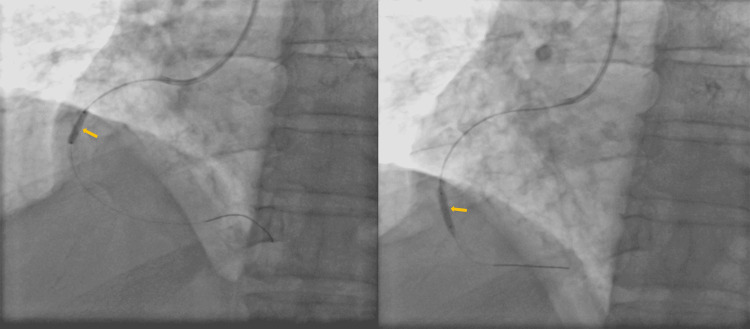
A 3.5 JR guiding catheter was engaged in RCA ostium and was crossed wire to distal RCA and sequentially IVL done RCA: right coronary artery; IVL: intravascular lithotripsy

The patient was diagnosed as having a single-vessel disease. A 3.5 JR guiding catheter (Medtronic, Minneapolis, MN, USA) was engaged in ostial RCA and then the guidewire was crossed to distal RCA. Thereafter, sequentially shockwave IVL was performed using a 2.0 × 10 mm balloon, which was inflated to 12 atmospheres, and 10 pulses of ultrasound energy of 10 seconds were successively delivered (Figure 3). This cycle was repeated four times.

**Figure 4:**
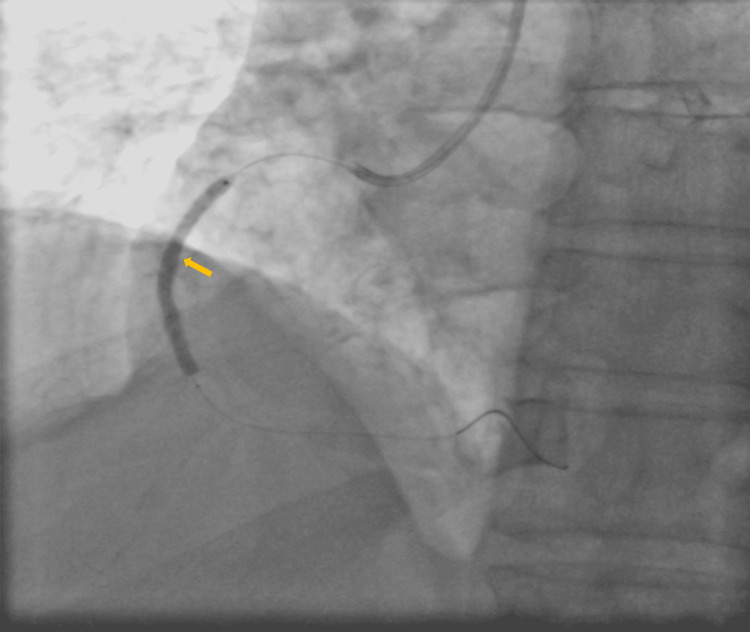
A 3.0 × 40 mm DES was negotiated and deployed DES: drug-eluting stent

**Figure 5:**
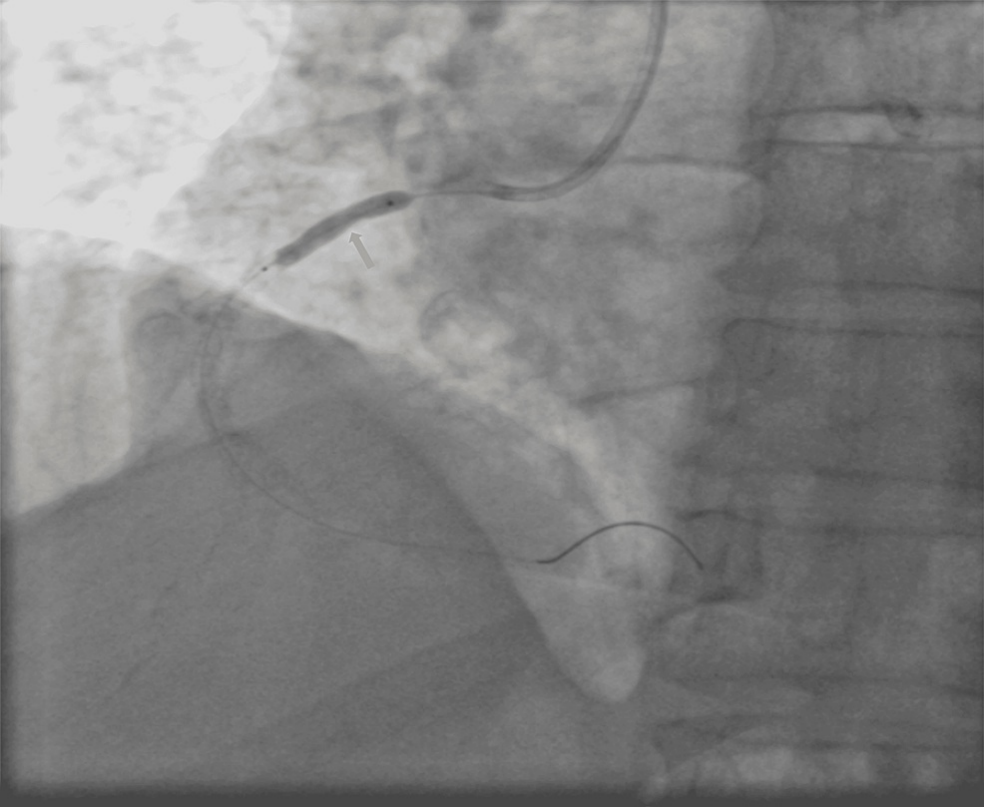
A 3.5 × 18 mm DES was deployed by overlapping with proximal of stent 3.0 × 40 mm DES: drug-eluting stent

**Figure 6:**
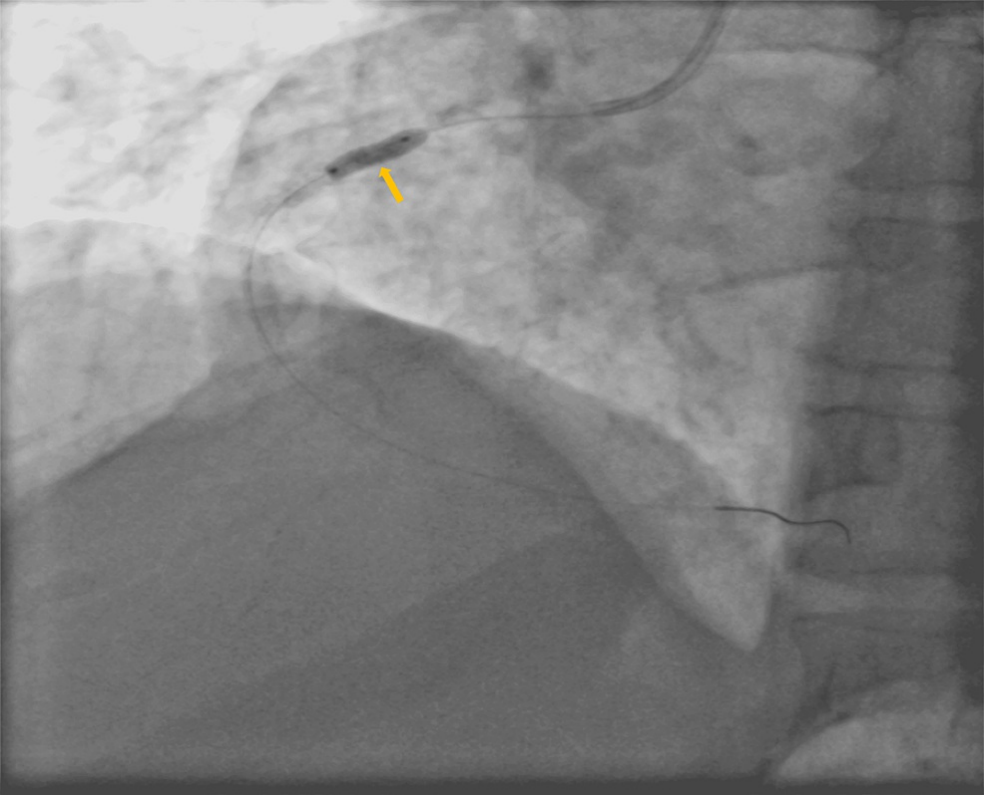
Sequentially post-dilated with a 3.5 × 10 mm non-compliant balloon

After shock wave IVL, two overlapped DESs of a 3.0 × 40 mm (Figure 4) and a 3.5 × 18 mm (Figure 5) sizes were implanted followed by post-dilatation with a 3.5 × 10 mm non-compliant balloon (Figure 6).

**Figure 7:**
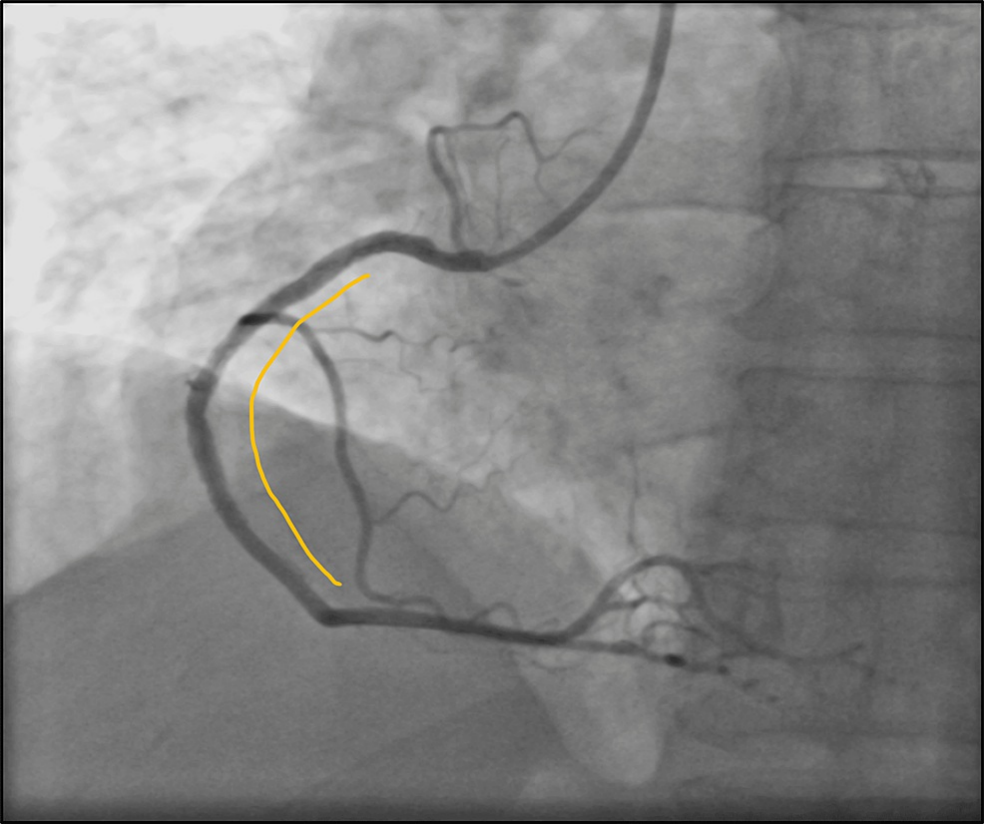
Final TIMI flow TIMI: thrombolysis in myocardial infarction

No complications occurred during hospitalization. After shockwave IVL-assisted staged PCI, excellent angiography outcomes have been achieved with the establishment of thrombolysis in myocardial infarction (TIMI) 3 flow (Figure 7).

## Discussion

The prevalence of CAC in PCI cases is estimated to be within the range of 18-26%; however, this number is expected to rise. Extremely calcified coronary lesions remain a hurdle for PCI as their dilatation and precise implantation of stents are quite challenging. Very tight calcified lesions may resist dilatation at low balloon inflation pressures or rupture at high pressures. This causes high rates of procedural complications and, consequently, poor clinical outcomes [6]. Recent therapeutic calcium debulking techniques used to overcome CAC can be categorized into two groups: non-balloon (rotational, orbital, and laser coronary atherectomy) and balloon-based technologies (high-pressure/ultra-high-pressure non-compliant balloon, scoring/cutting balloons, shockwave IVL balloon). Sadly, these techniques are encumbered by a significant number of procedural complications, such as distal embolization, vascular wall injury, and coronary artery dissection or perforation [7]. Derived from lithotripsy technology employed to treat nephrolithiasis [8], the shockwave IVL system has also proved to be beneficial in treating CAC. Compared to the aforementioned conventional approaches, the shockwave IVL system requires minimal training and provides excellent outcomes of luminal widening, successful stent implantation, and reduced risk of major adverse cardiovascular events [6]. These observations are noted in our case, too.

Disrupt coronary artery disease (CAD) II study, a prospective multicenter, single-arm post-approval study, concluded the safety and effectiveness of shock wave IVL-assisted PCI in severe CAC [9], this is in agreement with findings reported by Aksoy et al. [10]. Wong and coworkers [3] reported the usefulness of the shockwave IVL system in coronary calcium modification to optimize stent expansion in cases of acute coronary syndromes (ACS) (29% of ACS patients were staged PCIs for severe non-culprit lesions), stable angina, and PCI before transcatheter aortic valve implantation. According to Tsiafoutis et al. [11], the shock wave IVL-assisted PCI appears to be a safe and useful alternative to achieve procedural success in CAC in STEMI cases. Further, many authors have reported promising outcomes of shock wave IVL system in percutaneous revascularization of severely calcified LM disease [12], severely calcified and undilatable LAD lesions in a patient with recurrent myocardial infarction [4], chronic total occlusion (CTO) PCI [13].

All previously mentioned studies hold the view that shock wave IVL-assisted PCI remains the default strategy for severely calcified coronary stenoses. In the present case, calcified stenosis of RCA has been successfully treated with shockwave IVL-assisted staged PCI in patients presented with unstable angina. Considering the following facts, we have used shockwave IVL in our case: a) IVL has preferential effect on deep calcium than other ablation techniques, b) being a balloon-based technique, it is user-friendly with a short learning curve [14]. Patel and co-investigator [15] proposed the use of shockwave IVL in staged PCI during which thrombus burden and myocardial electrical instability may be significantly less. This notion is backed by the DEFER-STEMI (Deferred Stent Trial in STEMI), wherein deferring stent implantation in STEMI culminated in reduced no-reflow and increased myocardial salvage, with nearly 4% requiring urgent PCI prior to the staged procedure [16]. However, there is room for research on shockwave IVL-assisted staged PCI in treating CAC.

## Conclusions

CAC still represents one of the most challenging subsets in PCI because of worse clinical outcomes. Slowly but surely, the proportion of patients with severely calcified lesions has been projected to grow worldwide, including in India. The optimal treatment for the condition still remains demanding. IVL is a relatively novel technique designed to overcome calcified stenosis in coronary arteries, with promising outcomes from several clinical trials. In this case, our experience demonstrates that conjugation of shockwave IVL system with staged PCI is safe in treating CAC and associated with a low rate of complication and high procedural success.
